# Mammalian Cell Culture Process for Monoclonal Antibody Production: Nonlinear Modelling and Parameter Estimation

**DOI:** 10.1155/2015/598721

**Published:** 2015-01-19

**Authors:** Dan Selişteanu, Dorin Șendrescu, Vlad Georgeanu, Monica Roman

**Affiliations:** ^1^Department of Automation, Electronics and Mechatronics, University of Craiova, A.I. Cuza No. 13, 200585 Craiova, Romania; ^2^Faculty of General Medicine, Carol Davila University of Medicine and Pharmacy, Eroilor Sanitari No. 8, 050474 Bucharest, Romania

## Abstract

Monoclonal antibodies (mAbs) are at present one of the fastest growing products of pharmaceutical industry, with widespread applications in biochemistry, biology, and medicine. The operation of mAbs production processes is predominantly based on empirical knowledge, the improvements being achieved by using trial-and-error experiments and precedent practices. The nonlinearity of these processes and the absence of suitable instrumentation require an enhanced modelling effort and modern kinetic parameter estimation strategies. The present work is dedicated to nonlinear dynamic modelling and parameter estimation for a mammalian cell culture process used for mAb production. By using a dynamical model of such kind of processes, an optimization-based technique for estimation of kinetic parameters in the model of mammalian cell culture process is developed. The estimation is achieved as a result of minimizing an error function by a particle swarm optimization (PSO) algorithm. The proposed estimation approach is analyzed in this work by using a particular model of mammalian cell culture, as a case study, but is generic for this class of bioprocesses. The presented case study shows that the proposed parameter estimation technique provides a more accurate simulation of the experimentally observed process behaviour than reported in previous studies.

## 1. Introduction

As the market demand for monoclonal antibodies is increasing, there is significant interest in developing proper models for mammalian cell culture processes, due to the fact that these are commonly used as production platforms for mAbs, which are the fastest growing segment of the biopharmaceutical industry [1–6]. For mAb production, various mammalian cell lines are usually exploited, such as murine myeloma (NS0), murine hybridomas, Chinese hamster ovary (CHO), and PER.C6 human cells. The selection of expression system is determined by its capability to deliver high productivity with suitable product quality attributes [7]. Medical applications for mAbs are quite extensive: diagnostic tools, therapies for various cancers, rheumatoid arthritis, cardiovascular conditions, and so on [4, 6–9].

Typically, the industrial operation for mammalian cell culture mAb platforms relies on empirical knowledge [2, 3, 10] and the improvements are achieved by using trial-and-error experiments and precedent practices. Consequently, process improvements have generally been time-consuming and costly, with a high degree of specificity. To assist these laboratory experiments and, in practical terms, to achieve high productivity and better quality products, it is of obvious interest to develop model-based applications and to achieve accurate dynamical models. However, the specific characteristics of these processes, such as complexity, nonlinearity, and absence of cheap and reliable instrumentation, require an enhanced modelling effort and advanced kinetic parameter estimation strategies.

In order to surmount the above-mentioned limitations of trial-and-error process development, the so-called predictive models for mammalian cell culture processes are quite attractive [4]. Generically speaking, cell culture modelling techniques are classified based upon whether a dynamic or a pseudo-steady-state interpretation of cellular metabolism is used [2, 4, 11, 12]. Being well-known in control systems, the pseudo-steady-state approach has a biochemical interpretation in cell culture processes. It is assumed that all metabolites within the cell culture process are accumulated or depleted at a rate considerably faster than the overall cell growth rate. Consequently, the concentration of each system metabolite and the rate of each metabolic reaction are all considered time-invariant [4]. This approach is simple and the obtained models are linear systems, which can be easily computed regardless of the model size (complexity). The information gathered in such pseudo-steady-state models concerns the metabolic configuration of cell culture. However, mammalian cells have a complicated internal structure, with several interconnected biochemical processes and with phenomena on multiple time scales. Thus, the pseudo-steady-state models cannot describe in detail the changes that occur over a continuous time-horizon (intracellular concentration profiles, changes in reaction rate due to gene regulation, etc.). Therefore, the dynamic modelling is more appropriate for these complex (and dynamical) processes. In this case, a system of differential equations will describe the bioprocess model. In many cases, the difficulty that arises is related to the computational problems, especially for large and stiff systems. No matter what modelling method is chosen, the complexity together with the nonlinearity of these processes is a limiting factor in model building.

In this paper, which is an extended work of [13, 14], an essential problem in dynamic modelling of cell culture systems is analysed, the so-called parameter estimation. The model of such bioprocesses can be obtained by using dynamic classical modelling (based on mass balance) or alternative approaches such as pseudo-bond-graph method (a version of bond graph method introduced by Paynter in 1961 and further developed in [15–26]). However, regardless of the modelling method, in order to obtain a dynamical model useful for process development (including the design of some control strategies), the nonmeasurable parameters of the mammalian cell culture system must to be estimated. However, any parameter in a cell culture model could [4] have physical meaning and be measurable by experiment, have clear physical meaning but be experimentally inaccessible, or have no clear physical meaning (e.g., be purely mathematical in nature). Typically, optimization-based techniques are used for the estimation of nonmeasurable parameters of such biological processes [4, 27, 28]. For example, a quadratic programming technique was used by Gao et al. [27], and a simple discretization scheme combined with a filtered interior point primal-dual line-search algorithm [29] was proposed by Baughman et al. [4]. Other nonlinear optimization-based techniques are the genetic algorithms, orthogonal collocation, and particle swarm optimization, PSO, which have been applied mostly on chemical processes (see, e.g., [30, 31]) or in gene regulatory networks modelling [32, 33]. In order to obtain accurate solutions in the case of the mAb production process, in this paper, a particle swarm-based multistep nonlinear optimization algorithm is proposed [34–36].

Concerning the applications of PSO for identification of biological systems, some results were reported for the process of glycerol fermentation by* Klebsiella pneumoniae* in batch, fed-batch, and continuous cultures [37–41]. The estimation approach used in these works is in most cases a parallel PSO technique, which requires a considerable computational effort. Another trend is related to an indirect use of PSO technique for estimation, more precisely for the training of a neural network, which models the bioprocess [42].

During the last decade, PSO algorithms have gained much attention and wide applications in different fields due to their effectiveness in performing difficult optimization issues, as well as simplicity of implementation and ability of fast converge to a reasonably good solution. PSO is a population-based heuristic global optimization technique, first introduced by Kennedy and Eberhart [43] and referred to as a swarm-intelligence technique. It is motivated from the simulation of social behaviour of animals such as bird flocking, fish schooling, and swarm. In this algorithm, the population is called a swarm and the trajectory of each particle in the search space is controlled through the medium of a term called “velocity,” according to its own flying experience and swarm experience in the search space.

This paper proposes a multistep PSO version that uses time-varying acceleration coefficients [35], which is developed to solve the nonconvex optimization problem, ensuring fast convergence and very good performance. Finally, the obtained solution is an optimal set of the kinetic parameter values.

The proposed nonlinear modelling and estimation approaches are analyzed in this work by using a particular model of mammalian cell culture, as a case study, but they are generic for this class of bioprocesses. A previously published dynamic model of mammalian cell culture by Gao et al. in [27] is used as a case study. More precisely, a process of an Immunoglobulin G-secreting murine hybridoma cultured in a growth medium supplemented with proline, L-asparagine, and L-aspartic acid is taken into consideration.

## 2. Methods

### 2.1. MAb Synthesis by Mammalian Cell Culture: Process Description and Modelling Issues

In order to model the mammalian cell culture processes, first it is necessary to analyze the reconstruction of metabolic activities. However, the reconstruction generally includes only a subset of the highly active metabolic units found in proliferating mammalian cells [4, 34]. After the choice of this key subset, the next step consists in the modelling of the reactions' rates in the frame of reconstruction. This process is a very difficult one, and the modelling of reactions as single-enzyme processes by using* in vitro* kinetic parameters is possible only for simple and small reconstructions. Often,* in vitro* kinetic parameters do not compare well against* in vivo* observations [4]. The reconstruction in complex processes will frequently combine a number of discrete processes into a single lumped process and will then apply kinetic parameters to the lumped process. Consequently, some kinetic parameters that appear in the model may have small or no physical significance and usually their values are not experimentally measurable [4].

Therefore, if all of the interaction of metabolites and cell physiology are included in the modelling process, then the size of the obtained model is very large and it is not appropriate for model-based optimization and control purposes. The usual solution is to select a priori the elementary reaction schemes and to relate the major macroscopic species such as biomass, essential substrates, and products by a set of so-called macroreactions [27]. Thus, a simplified model is obtained, which is suitable for optimization and control. As was mentioned before, the next step in the modelling is related to the determination of reaction kinetics and the final model is obtained based on mass balance equations of the macroscopic species involved in the reactions.

Next, a particular model of mammalian cell culture, published by Gao et al. [27], will be described and used as a case study. Gao et al. [27] provided a detailed description of an Immunoglobulin G- (IgG-) secreting murine hybridoma (130-8F* Sanofi Pasteur*) cultured in a D-MEM (Dulbecco's Modified Eagle Medium) growth medium supplemented with proline, L-asparagine, and L-aspartic acid. In this process, batch cultures of the organism were allowed to grow for a minimum of 7 days. The infrequent measured concentration data for glucose, lactate, and ammonia, as well as for 20 amino acids and the monoclonal antibody, were obtained from the collected samples via proper techniques. By using the measured data, the average rates of transmembrane fluxes were calculated for each metabolite for both the initial exponential growth phase and for the postexponential (decline) phase. Gao et al. [27] used the metabolic flux analysis (MFA) in order to calculate the unknown intracellular fluxes from measured extracellular fluxes by applying steady-state mass balance equations. The obtained metabolic network was constructed based on some preliminary studies [44–47], and it represents the significant metabolic pathways in proliferating animal cells. Gao et al. [27] determined that 16 reactions (a half of the total number) in the chosen reconstruction did not function significantly, and consequently these reactions, with an activity of about 1% of the total, were eliminated. The remaining subset of 16 reactions of the reduced metabolic reconstruction was further reduced by using a technique that combines reactions that share common metabolites [48]. Finally, the reduced reaction scheme for this mAb bioprocess contains a number of only 11 extracellular compounds and it consists of nine macroreactions, presented in Table 1 [4, 27].

The dynamical model of a generic bioprocess inside a bioreactor can be obtained by using the mass balance of the component and it is given by the following set of differential equations [49]:

(1) $$\begin{array}{c} \frac{d\xi }{dt}=K\cdot \varphi \left(\xi \right)+D\cdot \xi +F-Q, \end{array}$$

where *ξ* = [*ξ*_1_ *ξ*_2_ ⋯ *ξ*_*n*_]^*T*^ is the *n*-dimensional vector of the instantaneous concentrations (the concentrations of extracellular metabolites in our particular case), *φ* = [*φ*_1_ *φ*_2_ ⋯ *φ*_*m*_]^*T*^ is the vector of the reaction rates, and *K* is the *n* × *m* dimensional matrix of stoichiometric (or yield) coefficients, with *K* = [*K*_*ij*_], $i=\bar{1,n}$; $j=\bar{1,m}$, where *K*_*ij*_ = (±)*k*_*ij*_ if *j* ~ *i*. The notation *j* ~ *i* indicates that the sum is done in accordance with the reactions *j* that involve the components *i*. The sign of the yield coefficients *k*_*ij*_ is given by the type of the component *ξ*_*i*_: plus (+) when the component is a reaction product and minus (−) otherwise. *D* is the specific volumetric outflow rate (h^−1^), usually called dilution rate. In (1), *F* = [*F*_1_ *F*_2_ ⋯ *F*_*n*_]^*T*^ is the vector of rates of liquid supply and *Q* = [*Q*_1_ *Q*_2_ ⋯ *Q*_*n*_]^*T*^ is the vector of rates of removal of the components in gaseous form.

Model (1) describes in fact the behaviour of an entire class of bioprocesses and is referred to as the general dynamical state-space model of this class [49, 50]. In (1), the term *K* · *φ*(*ξ*) is in fact the rate of consumption and/or production of the components in the reactor, that is, the reaction kinetics. The term −*Dξ* + *F* − *Q* represents the exchange with the environment. The strongly nonlinear character of this model is given by the reaction kinetics. In many practical situations the structure and the parameters of the reaction rates are partially known or even completely unknown.

Typically, in a batch process the reactor is filled with the reactant mixture: substrates and microorganisms. Then, the reactions occur inside the reactor for a time period; after that, the products are removed from the tank. Because the studied bioprocess takes place inside a batch reactor, model (1) becomes

(2) $$\begin{array}{c} \frac{d\xi }{dt}=K\cdot \varphi \left(\xi \right); \end{array}$$

that is, the term −*Dξ* + *F* − *Q* (which represents the exchange with the environment) is zero in this particular batch mode.

For the mAb production process, the concentrations of the 11 extracellular metabolites (given in the reaction scheme from Table 1) constitute the elements of the state vector from model (1) and are denoted as follows:

(3) $$\begin{array}{c} \xi =\left[\begin{array}{c} \xi_{1}\\ \xi_{2}\\ \xi_{3}\\ \xi_{4}\\ \xi_{5}\\ \xi_{6}\\ \xi_{7}\\ \xi_{8}\\ \xi_{9}\\ \xi_{10}\\ \xi_{11} \end{array}\right]=\left[\begin{array}{c} S_{1}\\ S_{2}\\ S_{3}\\ S_{4}\\ S_{5}\\ P_{1}\\ P_{2}\\ P_{3}\\ P_{4}\\ P_{5}\\ P_{6} \end{array}\right]=\left[\begin{array}{c} \text{GLC}\\ \text{GLN}\\ \text{GLU}\\ \text{ASN}\\ \text{ASP}\\ \text{LAC}\\ \text{ALA}\\ \text{PRO}\\ \text{MAb}\\ \text{BM}\\ {\text{NH}}_{3} \end{array}\right], \end{array}$$

where GLC = glucose, GLN = glutamine, GLU = glutamate, ASN = asparagine, ASP = aspartate, LAC = lactate, ALA = alanine, PRO = proline, MAb = monoclonal antibody, BM = biomass, NH_3_ = ammonia are the metabolites given in Table 1 (and for simplicity, the concentrations of the corresponding elements in model (1)).

However, in order to complete the model of the mAb production process, it is necessary to add the evolution of the viable cell concentrations of the culture, because the metabolite mass balances depend on the amount of viable cells. Gao et al. [27] noticed the typical behaviour of the batch culture, with exponential growth and postexponential decline, senescence phase (which occurs after the first phase of evolution, due to the aging of the cells and the accumulation of autoinhibitory metabolites). Therefore, another two concentrations enter in the complex model of the bioprocess, the viable cell concentration *X* and the dead cell concentration *X*_*d*_. The dynamics of these concentrations will be modelled separately, depending of the phase (growth or decay).

**Remark 1 .**

To be exact, for the mAb production process the exchange with environment is zero except the CO_2_ gaseous flow, but this flow is not measured and CO_2_ is not predicted in the final model, as it is considered in [27].

In the following, the dynamical model (2) of the mAb production process will be presented, starting with the reaction scheme given in Table 1. Afterward, the problem of kinetic rates is addressed, together with the parameter estimation problem, via PSO-based techniques.

The dynamical model of the form (2) can be particularized for the mAb production process described by the reaction scheme from Table 1 by using the mass balance of the components (via classical methods [4, 27] or bond graph approach [13]) inside the batch reactor. The following dynamical model is obtained:

(4) dS1dt=−φ1−φ2−φ3−k1,7φ7,dS2dt=−φ6−k2,7φ7−k2,8φ8−φ9,dS3dt=−k3,2φ2−k3,3φ3−φ4+φ6−k3,7φ7−k3,8φ8+φ9,dS4dt=−φ5+φ6−k4,7φ7−k4,8φ8,dS5dt=k5,3φ3+φ5−φ6−k5,7φ7−k5,8φ8,dP1dt=k6,1φ1+k6,2φ2+k6,3φ3,dP2dt=k7,2φ2−k7,7φ7−k7,8φ8,dP3dt=φ4−k8,7φ7−k8,8φ8,dP4dt=φ8,dP5dt=φ7,dP6dt=φ5+φ9.

Model (4) can be written in a compact form [13]:

(5) $$\begin{array}{c} \frac{d}{dt}\underset{\xi }{\underset{︸}{\left[\begin{array}{c} S_{1}\\ S_{2}\\ S_{3}\\ S_{4}\\ S_{5}\\ P_{1}\\ P_{2}\\ P_{3}\\ P_{4}\\ P_{5}\\ P_{6} \end{array}\right]}}=\underset{K}{\underset{︸}{\left[\begin{array}{ccccccccc} -1 & -1 & -1 & 0 & 0 & 0 & -k_{1,7} & 0 & 0\\ 0 & 0 & 0 & 0 & 0 & -k_{2,6} & -k_{2,7} & -k_{2,8} & -1\\ 0 & -k_{3,2} & -k_{3,3} & -1 & 0 & 1 & -k_{3,7} & -k_{3,8} & 1\\ 0 & 0 & 0 & 0 & -1 & 1 & -k_{4,7} & -k_{4,8} & 0\\ 0 & 0 & k_{5,3} & 0 & 1 & -1 & -k_{5,7} & -k_{5,8} & 0\\ k_{6,1} & k_{6,2} & k_{6,3} & 0 & 0 & 0 & 0 & 0 & 0\\ 0 & k_{7,2} & 0 & 0 & 0 & 0 & -k_{7,7} & -k_{7,8} & 0\\ 0 & 0 & 0 & 1 & 0 & 0 & -k_{8,7} & -k_{8,8} & 0\\ 0 & 0 & 0 & 0 & 0 & 0 & 0 & 1 & 0\\ 0 & 0 & 0 & 0 & 0 & 0 & 1 & 0 & 0\\ 0 & 0 & 0 & 0 & 1 & 0 & 0 & 0 & 1 \end{array}\right]}}\underset{\varphi (\xi )}{\underset{︸}{\left[\begin{array}{c} \varphi_{1}\\ \varphi_{2}\\ \varphi_{3}\\ \varphi_{4}\\ \varphi_{5}\\ \varphi_{6}\\ \varphi_{7}\\ \varphi_{8}\\ \varphi_{9} \end{array}\right]}}, \end{array}$$

where the values of stoichiometric coefficients are given in the reaction schemes from Table 1 and are as follows [4, 27]: *k*_1,7_ = 0.0508, *k*_2,6_ = 1, *k*_2,7_ = 0.0577, *k*_2,8_ = 0.0104, *k*_3,2_ = 2, *k*_3,3_ = 2, *k*_3,7_ = 0.0016, *k*_3,8_ = 0.0107, *k*_4,7_ = 0.006, *k*_4,8_ = 0.072, *k*_5,3_ = 2, *k*_5,7_ = 0.0201, *k*_5,8_ = 0.082, *k*_6,1_ = 2, *k*_6,2_ = 2, *k*_6,3_ = 2, *k*_7,2_ = 2, *k*_7,7_ = 0.0133, *k*_7,8_ = 0.011, *k*_8,7_ = 0.081, and *k*_8,8_ = 0.0148.

The nonlinear dynamical model (5) is obvious from the general form (2). However, in order to complete the model of mAb production process, it is necessary to add the submodels corresponding to the dynamics of viable cell concentration and dead cell concentration, respectively. Here it should be noted that Gao et al. [27] have obtained from the experimental observations that the model describing viable cell growth changes at *t* = 54 h to reflect the transition from exponential growth to the decline phase. With this remark, the dynamical model of the viable and cell concentrations evolutions is as follows [27]:

(6) dXdt=μX−kdXXd, for  t<texp⁡,dXdt=−kdXXd, for  t≥texp⁡,dXddt=kdXXd,

where *μ* is the specific growth rate of the viable cells, *k*_*d*_ is a kinetic (decay) parameter, and *t*_exp⁡_ is the time period of the exponential growth phase.

The most difficult modelling problem for the system of differential equations (5), (6) is related to the model of nonlinear reaction kinetics. Gao et al. [27] suggested that a generalized form of saturable kinetics (i.e., compound Monod kinetics) is suitable to describe the rate of each macroreaction from the reaction scheme given in Table 1. Rates for each of these macroreactions were expressed in the next compact form [4, 31]:

(7) $$\begin{array}{c} \varphi_{i}=\varphi_{i}^{*}\cdot X\cdot \overset{}{\underset{S_{j}\in S_{i}}{\prod }}\frac{S_{j}}{{K_{S}}_{j,i}+S_{j}},\;i=\bar{1,9}. \end{array}$$

In the kinetic rates expression (7), *φ*_*i*_ is the reaction rate for reaction *i*, *φ*_i_^*^ is the maximum reaction rate for reaction *i*, *S*_*j*_ is the concentration of substrate *j* within the set *S*_*i*_ of substrates for reaction *i*, and *K*_*S*_*j*,*i*__ is a kinetic half-saturation constant for substrate *j* in reaction *i*. The specific rate expressions for each macroreaction are given in Table 2 [4, 27]. As Baughman et al. [4] noticed, the rate expressions for macroreactions 7 and 8 do not rigorously conform to the general format (7). More precisely, it was assumed that the principal rate-limiting substrate for both biomass and antibody synthesis is glutamine, and the kinetic contributions of any other substrates were thus omitted.

In conclusion, the full dynamical model of mAb production process is given by (5), where the kinetic rates are of the form presented in Table 3, together with the dynamical models (6) of viable and dead cell evolution in the batch reactor.

The state variables within the dynamical model (5)–(7) are associated with components of the macroreactions from the reaction scheme given in Table 1. While these components represent biological variables (concentrations of some substances or compounds), the kinetic parameters do not have always clear measurable physical representations.

The problem that remains to be solved now is related to the estimation of the unknown (inaccessible) kinetic parameters of the dynamical model (5), (6) of the mammalian cell culture. Therefore, it is necessary to estimate the experimentally inaccessible parameter values for the model that provide the best approximation to the measured culture concentrations data.

### 2.2. PSO-Based Technique Parameter Estimation

#### 2.2.1. Problem Statement and Basic PSO Algorithms

At the beginning of parameter estimation, the input and output data are known and the real system parameters are assumed as unknown. The identification problem is formulated in terms of an optimization problem in which the error between an actual physical measured response of the system and the simulated response of a parameterized model is minimized. The estimation of the system parameters is achieved as a result of minimizing the error function by the PSO algorithm.

Consider that the nonlinear system (2) that describes the dynamical behaviour of a class of bioprocesses is written as the following *n*-dimensional nonlinear system:

(8) $$\begin{array}{c} \frac{d\xi }{dt}=K\cdot \varphi \left(\xi \right)=f\left(\xi ,t;\theta \right), \end{array}$$

where *ξ* ∈ *R* ^*n*^ is the state vector (i.e., the vector of concentrations), *θ* ∈ *R* ^*q*^ is the unknown parameters vector (i.e., the vector of unknown kinetic parameters), and *f* is a given nonlinear vector function.

To estimate the unknown parameters in (8), a parameter identification system is defined as follows:

(9) $$\begin{array}{c} \frac{d\hat{\xi }\left(t\right)}{dt}=f\left(\hat{\xi },t;\hat{\theta }\right), \end{array}$$

where $\hat{\xi }\in R^{n}$ is the estimated state vector and $\hat{\theta }\in R^{q}$ is the estimated parameters vector.

The objective function defined as the mean squared errors between real and estimated responses for a number *N* of given samples is considered as fitness of estimated model parameters [14]:

(10) $$\begin{array}{c} W=\frac{1}{N+M}\overset{M}{\underset{j=1}{\sum }}\overset{N}{\underset{k=1}{\sum }}{\left(\xi_{j}^{k}-{\hat{\xi }}_{j}^{k}\right)}^{2}, \end{array}$$

where *M* is the number of measurable states and *N* is the data length used for parameter identification, whereas *ξ*_*j*_^*k*^ and ${\hat{\xi }}_{j}^{k}$ are the real and estimated values of state *j* at time *k*, respectively.

This objective function is a function difficult to minimize because there are many local minima and the global minimum has a very narrow domain of attraction. Our goal is to determine the system parameters, using particle swarm optimization algorithms in such a way that the value of *W* is minimized, approaching zero as much as possible.

Mathematical description of basic PSO and some important variants is presented in the following.

Candidate solutions of a population called particles coexist and evolve simultaneously based on knowledge sharing with neighbouring particles. Each particle represents a potential solution to the optimization problem and it has a fitness value decided by optimal function. Supposing search space is *M*-dimensional, each individual is treated as a particle in the *M*-dimensional search space. The position and rate of position change for *i*th particle can be represented by *M*-dimensional vector, *x*_*i*_ = (*x*_*i*1_, *x*_*i*2_,…, *x*_*iM*_) and *v*_*i*_ = (*v*_*i*1_, *v*_*i*2_,…, *v*_*iM*_), respectively. The best position previously visited by the *i*th particle is recorded and represented by *p*_*i*_ = (*p*_*i*1_, *p*_*i*2_,…, *p*_*iM*_), called *pbest*. The swarm best position previously visited by all the particles in the population is represented by *p*_*g*_ = (*p*_*g*1_, *p*_*g*2_,…, *p*_*gM*_), called *gbest*. Then particles search their best position, which are guided by swarm information *p*_*g*_ and their own information *p*_*i*_. Each particle modifies its velocity to find a better solution (position) by applying its own flying experience (i.e., memory of the best position found in earlier flights) and the experience of neighbouring particles (i.e., the best solution found by the population). Each particle position is evaluated by using fitness function and updates its position and velocity according to the following equations:

(11) $$\begin{array}{c} v_{i}^{k+1}=\omega \cdot v_{i}^{k}+c_{1}r_{1}\left(pbest_{i}^{k}-x_{i}^{t}\right)+c_{2}r_{2}\left(gbest_{i}^{k}-x_{i}^{k}\right),\\ x_{i}^{k+1}=x_{i}^{k}+v_{i}^{k+1}, \end{array}$$

where *k* is iteration number, *ω* is inertia weight, *c*_1_ and *c*_2_ are two acceleration coefficients regulating the relative velocity toward local and global best position, and *r*_1_ and *r*_2_ are two random numbers from the interval [0,1]. Many effects have been made over the last decade to determinate the inertia weight. Various studies have shown that under certain conditions convergence is guaranteed to a stable equilibrium point [51]. These conditions include *ω* > (*c*_1_ + *c*_2_)/2 − 1 and 0 < *ω* < 1. The technique originally proposed was to bound velocities so that each component of *v*_*i*_ is kept within the range [*V*_min⁡_, *V*_max⁡_].

Unfortunately, this simple form of PSO suffers from the premature convergence problem, which is particularly true in complex problems since the interacted information among particles in PSO is too simple to encourage a global search. Many efforts have been made to avoid the premature convergence. One solution is the use of a constriction factor to insure convergence of the PSO, introduced in [45]. Thus, the expression for velocity has been modified as

(12) $$\begin{array}{c} v_{i}^{k+1}=h\cdot \left[v_{i}^{k}+c_{1}r_{1}\left(pbest_{i}^{k}-x_{i}^{t}\right)+c_{2}r_{2}\left(gbest_{i}^{k}-x_{i}^{k}\right)\right],\\ x_{i}^{k+1}=x_{i}^{k}+v_{i}^{k+1}, \end{array}$$

where *h* represents the constriction factor and is defined as

(13) $$\begin{array}{c} h=\frac{2}{\left(\left|2-\alpha -\sqrt{\alpha^{2}-4\alpha }\right|\right)},\;\alpha =c_{1}+c_{2}>4. \end{array}$$

In this variant of the PSO algorithm, *h* controls the magnitude of the particle velocity and can be seen as a dampening factor. It provides the algorithm with two important features [52]. First, it usually leads to faster convergence than standard PSO. Second, the swarm maintains the ability to perform wide movements in the search space, even if convergence is already advanced but a new optimum is found. Therefore, the constriction PSO has the potential to avoid being trapped in local optima while possessing a fast convergence. It was shown to have superior performance compared to a standard PSO [53].

It is shown that a larger inertia weight tends to facilitate the global exploration and a smaller inertia weight achieves the local exploration to fine-tune the current search area. The best performance could be obtained by initially setting *ω* to some relatively high value (e.g., 0.9), which corresponds to a system where particles perform extensive exploration, and gradually reducing *ω* to a much lower value (e.g., 0.4), where the system would be more dissipative and exploitative and would be better at homing into local optima. In [54], a linearly decreased inertia weight *ω* over time is proposed, where *ω* is given by the following equation:

(14) $$\begin{array}{c} \omega =\left(\omega_{i}-\omega_{f}\right)\cdot \frac{\left(k_{\max}-k\right)}{k_{\max}}+\omega_{f}, \end{array}$$

where *ω*_*i*_, *ω*_*f*_ are starting and final values of inertia weight, respectively, *k*_max⁡_ is the maximum number of the iteration, and *k* is the current iteration number. It is generally taken that starting value is *ω*_*i*_ = 0.9 and final value is *ω*_*f*_ = 0.4.

On the other hand, in [35] PSO was introduced with time-varying acceleration coefficients. The improvement has the same motivation and similar techniques as the adaptation of inertia weight. In this case, the cognitive coefficient *c*_1_ is decreased linearly and the social coefficient *c*_2_ is increased linearly over time as follows:

(15) $$\begin{array}{c} c_{1}=\left(c_{1f}-c_{1i}\right)\cdot \frac{\left(k_{\max}-k\right)}{k_{\max}}+c_{1i},\\ c_{2}=\left(c_{2f}-c_{2i}\right)\cdot \frac{\left(k_{\max}-k\right)}{k_{\max}}+c_{2i}, \end{array}$$

where *c*_1*i*_ and *c*_2*i*_ are the initial values of the acceleration coefficients *c*_1_ and *c*_2_ and *c*_1*f*_ and *c*_2*f*_ are the final values of the acceleration coefficients *c*_1_ and *c*_2_, respectively. Usually, *c*_1*i*_ = 2.5; *c*_2*i*_ = 0.5; *c*_1*f*_ = 0.5; and *c*_2*f*_ = 2.5 [14, 35, 36].

The dynamical model of mAb production process given by the relations (5), (6), associated with the expressions of the kinetic rates presented in Table 3, contains a number of 23 kinetic parameters (maximum reaction rates and kinetic half-saturation constants). In order to estimate these unknown (inaccessible) kinetic parameters of the complex dynamical model of mammalian cell culture, the measured concentrations are used and a PSO-based algorithm is implemented. The goal is to obtain a model that approximates as well as possible the behaviour of the process (expressed by means of the experimentally obtained data). The model of mAb production process under investigation is in fact based on several macroreactions; therefore it results in the fact that the kinetic parameters do not have always clear measurable physical representations. Thus, an optimization-based estimation technique is suitable for this set of kinetic parameters.

#### 2.2.2. Implementation of PSO-Based Technique

In the following, a multistep PSO-based version that uses time-varying acceleration coefficients is implemented and an optimal set of kinetic parameter values of the mAb production process is obtained.

In order to implement the PSO-based technique, the model of mAb production process (5), (6) obtained from the macroreactions schemes is used, translated into the generic parameter identification system represented in (9).

The experimental concentration values for all the involved extracellular metabolites are provided in the work of Gao et al. [27]. The batch cultures of the organism were allowed to grow for 147 h, with 54 h the exponential growth phase and 93 h the postexponential (decline) phase. The infrequent measured concentration data for the metabolites were obtained from the collected samples via proper techniques. The set of concentrations measurements are given in Table 3 [4, 27]. Each data point is the average of measurements taken from three independent experiments, with standard deviation [4, 27].

To facilitate the application of the proposed PSO-based parameter estimation strategy, the time derivatives of the states from model (5)–(7) must be reconstructed. Because the measured data are very few (only 7 experimental measurements for each parameter, see Table 3), an interpolation method is necessary to find intermediate values of the states, which are actually the biological parameters of the process, that is, the concentrations of metabolites. Such situations with a small number of experimental measurements are typical for many bioprocesses. Ideally speaking, the online measurements (in each sampling moment, at every 6 min., e.g.) for each concentration are necessary. However, these online measurements are achieved with expensive instrumentation, or there are no such fast sensors for some concentrations. Thus, the infrequent offline measurements are preferred. To facilitate the achievement of an accurate estimation of model parameters of mAb process, we need the interpolation of these measured data, which allows us to obtain the unavailable data between adjacent measurement points (i.e., to estimate the unavailable data needed to calculate model predictions between these measurement points).

**Remark 2 .**

From mathematical point of view, a discussion about the interpolation technique can be done. Many authors use the linear interpolation, with advantages related to rapidity and simple implementation [4]. However, the linear interpolation is not very precise. Another disadvantage is that the interpolant is not differentiable at the points where the value of the function is known. Therefore, we propose a cubic interpolation method that is the simplest method that offers true continuity between the measured data. A cubic Hermite spline or cubic Hermite interpolator is a spline where each piece is a third-degree polynomial specified in Hermite form, that is, by its values and first derivatives at the end points of the corresponding domain interval. Cubic Hermite splines are typically used for interpolation of numeric data specified at given argument values *t*_1_, *t*_2_,…, *t*_*n*_, to obtain a smooth continuous function. The Hermite formula is applied to each interval (*t*_*k*_, *t*_*k*+1_) separately. The resulting spline will be continuous and will have continuous first derivative.

The time derivatives of the states are approximated using forward differences:

(16) $$\begin{array}{c} \frac{d\xi \left(t_{k}\right)}{dt}\approx \frac{\xi \left(t_{k}\right)-\xi \left(t_{k}+T_{s}\right)}{T_{s}}, \end{array}$$

where *T*_*s*_ represents the sampling period. In this approximation, the error is proportional with the sampling interval (a smaller sampling period will give a smaller approximation error).

**Figure 1:**
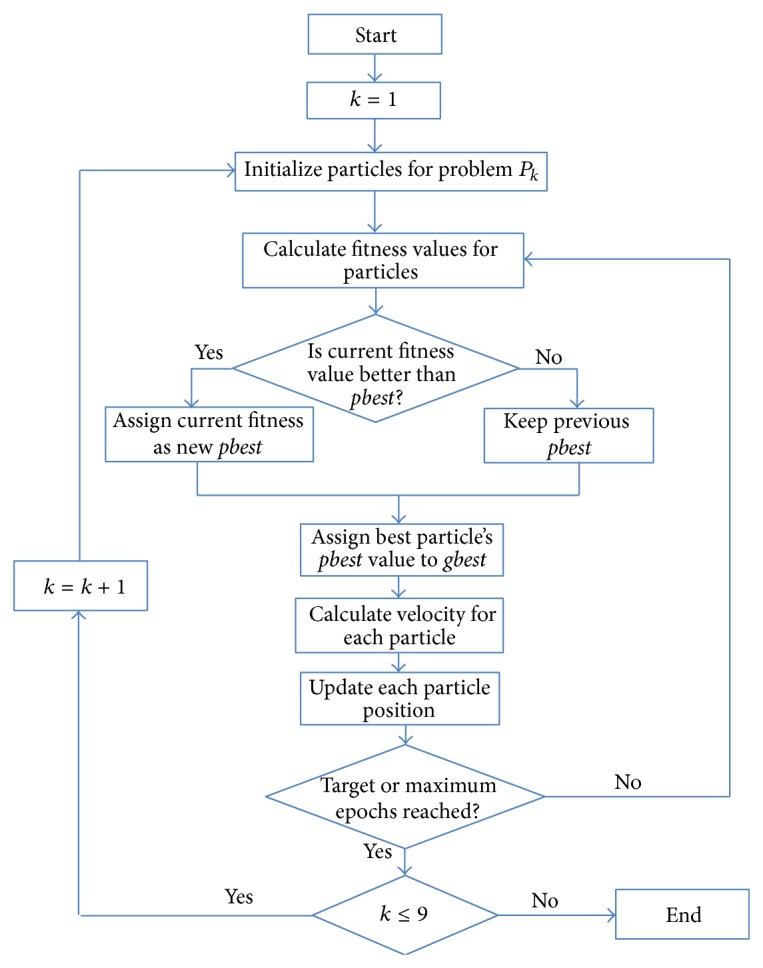
The flowchart of the multistep PSO algorithm.

Because a 23-dimensional optimization problem that must be solved for simultaneously estimation of all unknown parameters requires great computational resources, a multistep approach was used. So, the problem was split in nine simpler problems that are solved sequentially until all 23 parameters are found. These problems are noted with *P*1, *P*2,…, *P*9 and the corresponding resulted parameters are presented in Table 4. A flowchart of the multistep PSO algorithm is given in Figure 1.

For example, the problem *P*1 corresponds to the 10th equation from system (5)-(6) (that represents time evolution of the biomass) and only two parameters must be estimated in this case: *φ*_7_^*^ and *K*_*S*_2,7__. The PSO algorithm is used to minimize the sum of the square errors between measured and estimated data:

(17) WP1=∑k=1Nξ10(k·Ts)−ξ^10(k·Ts)2s.t. ξ^10k+1·Ts  =ξ^10k·Ts+Ts·φ7∗·ξ2k·Ts·ξ12k·TsKS2,7+ξ2k·Ts.

## 3. Results and Discussion

### 3.1. Optimal Set of Kinetic Parameters

The optimization problem formulated in the previous section is nonlinear and nonconvex with many local minima. The estimated parameters of one subproblem are then considered known in the subsequent equations. In order to be clear, the already estimated parameters are not updated between solutions of subproblems. For example, in the frame of problem *P*1 two parameters are estimated: *φ*_7_^*^ and *K*_*S*_2,7__. These parameters will be available in the next problem (*P*2), and so on. In this study, all the computations were achieved with a sampling period *T*_*s*_ = 6 min (0.1 h). As example, for problem *P*1 a number of 150 particles randomly initialized were used. The algorithm stops if the square error is smaller then 10*e* − 6 or after 300 iterations. The optimal set of kinetic parameter values of the mAb production process obtained via this multistep PSO-based approach is given in Table 5.

The partition of the multidimensional optimization problem proposed within our PSO algorithm not only ensures the decrease of necessary computational resources by comparison with Gao et al. [27] and Baughman et al. [4] approaches but additionally offers a solution for the reported problems concerning the stiffness of estimated parameter set. More precisely, as Baughman et al. [4] noticed, there are some concentrations such as the mAb concentration that are several orders of magnitude less than other metabolites. This fact leads to stiffness problems in the optimization procedure, which are partially solved in [4] by using an alternative error objective for the mAb concentration. In our approach, the partition in simpler PSO problems solves uniformly this issue, using the same error objective for the entire set of parameters.

**Figure 2:**
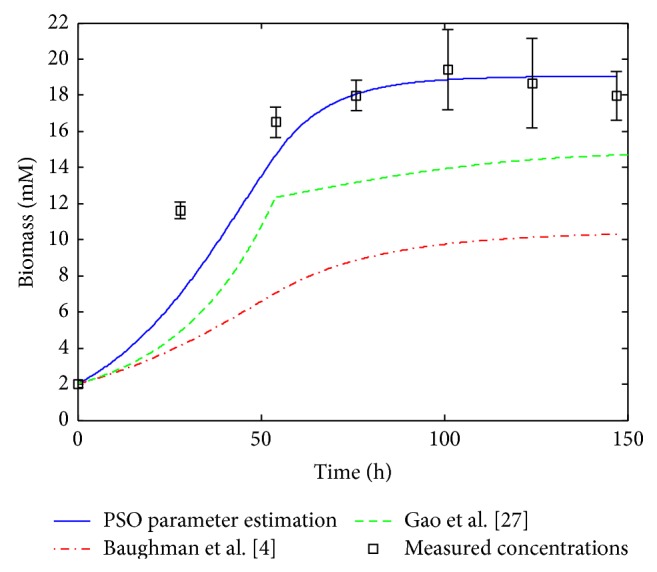
Simulation results, profile of the biomass concentration.

**Figure 3:**
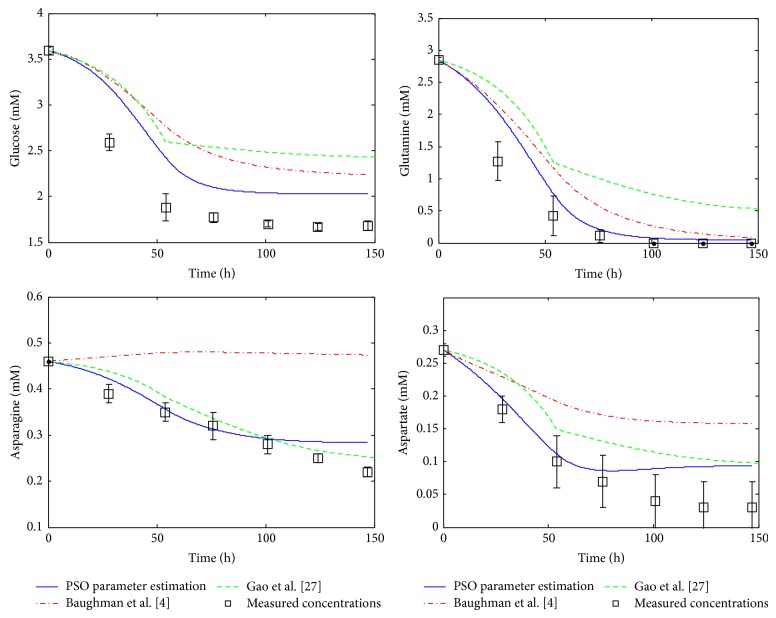
Simulation results, profiles of substrate concentrations: GLC, GLN, ASN, and ASP.

**Figure 4:**
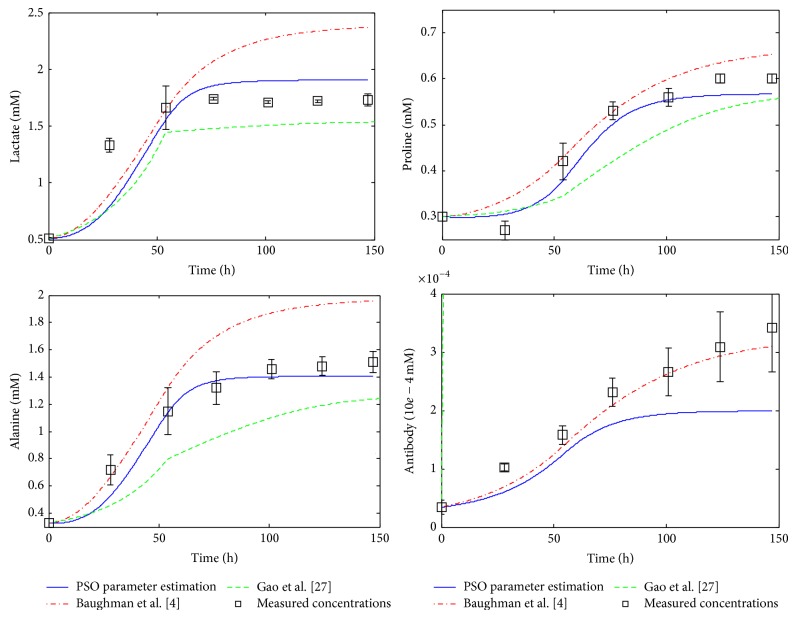
Simulation results, profiles of concentrations: LAC, PRO, ALA, and MAb.

**Figure 5:**
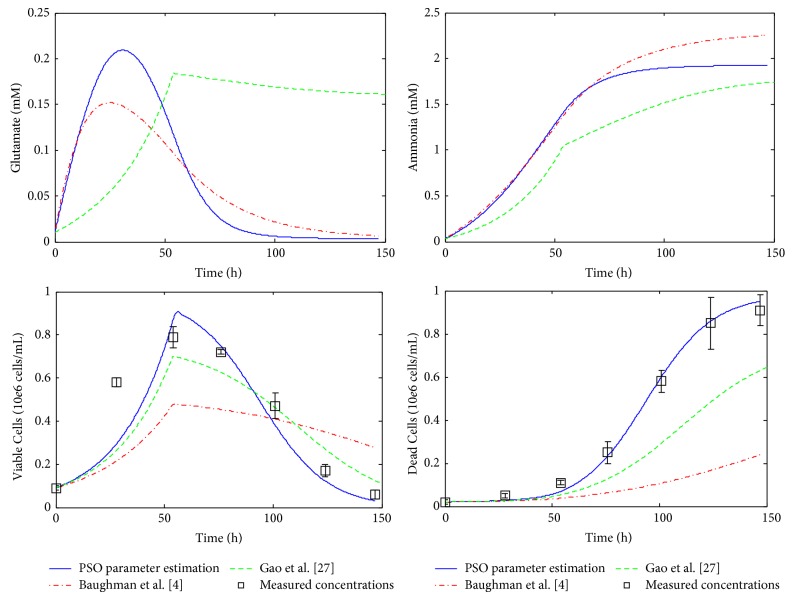
Simulation results, profiles of concentrations: GLU, NH_3_, X, and Xd.

Another important problem approached and solved by using the proposed PSO method is related to the expressions of reaction kinetics. To simplify the optimization problem, Gao et al. [27] considered that the values of half-saturation constants are sufficiently small and consequently the kinetic rates given in Table 2 can be simplified such that the relation (7) becomes *φ*_*i*_ = *φ*_i_^*^ · *X*, $i=\bar{1,9}$. This simplification eliminates the necessity of half-saturation constants estimation, and only the maximum reaction rates need to be estimated. However, as was mentioned in [4], half-saturation constants can be significantly smaller than the corresponding substrate concentration in processes controlled by a single enzyme (e.g., glucose transport), but this fact is not necessarily true for the macroreactions in mAb production processes. Therefore, Gao et al. assumption is unwarranted and it can affect the reliability of the model. This is one of the reasons because the proposed PSO method yield good fitting results compared with Gao et al. [27], as it can be seen in Figures 2–5.

**Remark 3 .**

There are necessary some comments concerning the overfitting problems. Overfitting arises when a statistical model describes noise instead of the underlying relationship; it usually occurs when a model is very complex, such as having many parameters relative to the number of observations. Even though the approached mAb production model is quite complex, it is not a statistical model. Also, even if the number of measured concentration samples is relatively small by comparison with the number of parameters, the PSO technique is an optimization procedure, which is much less sensitive to overfitting than the methods that are based on model training, such as neural network techniques (see Tetko et al. [55]). The potential for overfitting depends not only on the number of parameters and measured data but also on the conformability of the model structure with the data shape.

Some comparisons of the proposed PSO approach with other PSO applications to bioprocesses can be done. Most of the reported works were focused on the process of glycerol fermentation by* Klebsiella pneumoniae* in batch, fed-batch, and continuous cultures [37–41]. Shen et al. [37] studied a mathematical model of* Klebsiella pneumoniae* in a continuous culture. An eight-dimensional nonlinear dynamical system was obtained, and a parallel PSO technique was implemented in order to identify 19 parameters. The identification results are compared only with experimental steady-state values. The reported mean relative errors between the computational values and the experimental data are quite large (between 8% and 13%). A similar model of bio-dissimilation of glycerol by* K. pneumoniae* in a continuous culture was widely analyzed by Zhai et al. in [38]. Here a parallel PSO pathways identification algorithm was constructed to find the optimal pathway and 21 parameters under various conditions. The combined estimation of pathways and process parameters leads to a vast identification model, solved on a cluster server with 16 nodes (each node with 4 Core, 64-bit, 2.5 GHz processor), in over 130 hours. Comparable PSO approaches were used in [39, 41] in the case of the same fermentation process, but in a batch culture. For example, Yuan et al. [39] used a parallel migration PSO algorithm to estimate pathways and 12 parameters of the eight-dimensional model. The identification problem was split into two subproblems (one for pathways and one for parameters) and solved on the above cluster in about 18 hours. Another work addressed the PSO identification in the case of the fermentation of glycerol by* K. pneumoniae* in a fed-batch culture [40]. A nonlinear hybrid system was developed (with seven differential equations plus a switching mechanism and 8 parameters). The proposed technique was an asynchronous parallel PSO, and the reported averaged computational time was about 3.26 h, on the above-mentioned cluster server with 16 nodes. The reported results in [38–40] are very good, even the accuracy of the algorithms cannot be fully assessed (statistical reports were not provided). However, the computational effort is considerable, given the fact that simultaneous pathways and parameters identification was approached.

The particle swarm-based multistep nonlinear optimization algorithm proposed in the present work was used for the estimation of 23 parameters of an eleven-dimensional nonlinear system (the pathways identification was not considered). By using the multistep approach, the computational effort was quite small (about 20 min. on a computer with Intel Core i5, 64-bit, 3.3 GHz processor). The obtained results and the statistical analysis show a good accuracy of the identification results.

### 3.2. Simulation Results

The performance of the proposed estimation technique was analyzed by using numerical simulations. All these simulations are achieved by using the development, programming, and simulation environment MATLAB (registered trademark of The MathWorks, Inc., USA). For comparison, the simulated profiles based on the kinetic parameters obtained via PSO technique (Table 5) are represented together with the original system measurements [27] and with the profiles obtained by Gao et al. [27] and Baughman et al. [4], respectively. The concentration profiles based on the results of Gao et al. and on the results of Baughman et al. [4] approach, respectively, are simulated and plotted using the kinetic parameter values given in Table 6 [4, 27].

The simulated concentration profiles are presented in Figures 2–5. First, in Figure 2 the time evolution of the biomass concentration is depicted. As can be seen, the best matching with the measured data (the values of measured data plotted in all figures are taken from Table 3) is given by the PSO approach.  Figure 3 presents the simulated concentration profiles of glucose, glutamine, asparagine, and aspartate. In all cases, the PSO proposed technique ensures the best estimates (in the case of asparagine, the results of Gao et al. [27] are comparable with those obtained using PSO). In Figure 4, the concentration profiles of lactate, proline, alanine, and monoclonal antibody are plotted. The best matching with the measured data is obtained with the PSO estimation technique for lactate, proline, and alanine. In the case of antibody, the best results were obtained by Baughman et al. [4]. Finally, Figure 5 shows the concentration evolutions of glutamate, ammonia, viable, and dead cells, respectively. The glutamate and ammonia concentrations are not measured; the time profiles of these variables were obtained from the dynamic model simulation. The viable cells and dead cells evolution obtained via PSO estimation match very well the measured data.

Since in industrial practice the measured data are affected by various disturbances, one explored the extent to which noisy measurements affects the estimated parameter values. For this reason, a Monte Carlo simulation approach was used. First, normal (Gaussian) distributions were constructed for every measured data set in Table 3, subject to the known mean and standard deviation of each point.

A set of 150 simulated measurement sets were generated. Finally, using each randomized data set, a new cubic interpolation of the data was generated for our standard condition and the parameter estimation problems (*P*1 − *P*9) were solved for each case.

Outlying solutions were identified and excluded using a basic quartile classification method. The quartile values are chosen in the following manner. First, use the median to divide the ordered data set into two halves. The median is not included into the halves. Then, the lower quartile value is the median of the lower half of the data (*q*_low_) and the upper quartile value is the median of the upper half of the data (*q*_up_). Namely, the lower and upper quartile (*q*_low_, *q*_up_) positions were found for the set of 150 objective values and the interquartile range *q*_up_ − *q*_low_ was calculated. Solutions with objective values lying outside the interval [*q*_low_ − 1.5(*q*_*up*_ − *q*_low_), *q*_*up*_ + 1.5(*q*_up_ − *q*_low_)] were considered to be outlying cases. Using the remaining solutions, mean values and associated standard deviations were calculated for each estimated parameter.

The standard deviations were then converted to percentages of their associated mean value. These means and standard deviations are listed in Table 7.

Certain parameter estimates are much more susceptible to variability induced through perturbations in measured data than are others. It can be seen that certain parameters (*μ*, *k*_*d*_, *K*_*S*_1,3__) are less sensitive to noisy measurements than are certain others (*φ*_7_^*^, *K*_*S*_2,6__, *K*_*S*_2,8__, *K*_*S*_5,6__).

The proposed modelling and parameter estimation method can be applied to cellular processes described by the general form (1), and it is not yet applicable to all classes of bioprocesses. To be more specific, it is hard to be applied to processes characterized by phenomena such as propagation reactions, transport processes, latency and short intercellular phases (in epidemics), and spread (propagation) of infections, that is, processes with large heterogeneity and delays. A typical class of such nonlinear delay biosystems is represented by the dynamics models describing cell-to-cell spread mechanisms, encountered, for example, in HIV infections [56, 57].

## 4. Conclusions

In order to develop accurate models for mammalian cell culture processes and to overcome some of the specific problems of mAb production processes such as the nonlinearity, the absence of instrumentation, and the kinetics uncertainties, a multistep nonlinear particle swarm optimization-based technique for the estimation of experimentally unavailable kinetic parameters was designed and implemented. The proposed approach was tested by using a particular dynamical model of mammalian cell culture, as a case study, but is generic for this class of bioprocesses. We have established the capability of proposed technique to identify model parameters that provide an accurate simulation of experimentally observed mAb production process behaviour. The performed statistical analysis demonstrates that the proposed estimation method is robust against normal distributed noisy measurements. The simulations showed that the PSO parameter estimation technique provides more accurate results than those reported in previous studies.

The obtained dynamical model of the mAb production process is accurate and can contribute to the development of model-based applications, which lead to high productivity and better quality products. The performed simulations represent one of the possibilities of model validation. The results show that the proposed model offers good predictions not only of the cell culture, for instance predictions of concentrations of energy sources such as glutamine and glucose, but also of the main amino acids and products. The proposed estimation approach can be also applied to other bioprocesses belonging to the nonlinear class considered in the present study.

## Figures and Tables

**Table 1:** Macroreactions of the mAb production process [4, 27].

Reaction number	Macroreaction scheme
1	GLC → 2LAC
2	GLC + 2GLU → 2ALA + 2LAC
3	GLC + 2GLU → 2ASP + 2LAC
4	GLU → PRO
5	ASN → ASP + NH_3_
6	GLN + ASP → ASN + GLU
7	0.0508GLC + 0.0577GLN + 0.0133ALA + 0.006ASN + 0.0201ASP + 0.0016GLU + 0.081PRO → BM
8	0.0104GLN + 0.011ALA + 0.072ASN + 0.082ASP + 0.0107GLU + 0.0148PRO → MAb
9	GLN → GLU + NH_3_

**Table 2:** Kinetics expressions for the macroreactions [4, 27].

Reaction number	Kinetic rate
1	$\varphi_{1}=\varphi_{1}^{*}X\frac{S_{1}}{K_{S_{1,1}}+S_{1}}$
2	$\varphi_{2}=\varphi_{2}^{*}X\frac{S_{1}}{K_{S_{1,2}}+S_{1}}\frac{S_{3}}{K_{S_{3,2}}+S_{3}}$
3	$\varphi_{3}=\varphi_{3}^{*}X\frac{S_{1}}{K_{S_{1,3}}+S_{1}}\frac{S_{3}}{K_{S_{3,3}}+S_{3}}$
4	$\varphi_{4}=\varphi_{4}^{*}X\frac{S_{3}}{K_{S_{3,4}}+S_{3}}$
5	$\varphi_{5}=\varphi_{5}^{*}X\frac{S_{4}}{K_{S_{4,5}}+S_{4}}$
6	$\varphi_{6}=\varphi_{6}^{*}X\frac{S_{2}}{K_{S_{2,6}}+S_{2}}\frac{S_{5}}{K_{S_{5,6}}+S_{5}}$
7	$\varphi_{7}=\varphi_{7}^{*}X\frac{S_{2}}{K_{S_{2,7}}+S_{2}}$
8	$\varphi_{8}=\varphi_{8}^{*}X\frac{S_{2}}{K_{S_{2,8}}+S_{2}}$
9	$\varphi_{9}=\varphi_{9}^{*}X\frac{S_{2}}{K_{S9}+S_{2}}$

**Table 3:** Experimental concentration measurements [4, 27].

Time	0 h	28 h	54 h	76 h	101 h	124 h	147 h
GLC [mM]	3.59 ± 0.04	2.59 ± 0.09	1.88 ± 0.15	1.77 ± 0.05	1.70 ± 0.03	1.67 ± 0.04	1.68 ± 0.05
GLN [mM]	2.85 ± 0.04	1.27 ± 0.30	0.42 ± 0.31	0.11 ± 0.10	0.00 ± 0.00	0.00 ± 0.00	0.00 ± 0.00
ASN [mM]	0.46 ± 0.00	0.39 ± 0.02	0.35 ± 0.02	0.32 ± 0.03	0.28 ± 0.02	0.25 ± 0.02	0.22 ± 0.02
ASP [mM]	0.27 ± 0.02	0.18 ± 0.02	0.10 ± 0.04	0.07 ± 0.04	0.04 ± 0.04	0.03 ± 0.04	0.03 ± 0.04
LAC [mM]	0.51 ± 0.01	1.33 ± 0.06	1.66 ± 0.19	1.74 ± 0.01	1.71 ± 0.01	1.72 ± 0.01	1.73 ± 0.05
ALA [mM]	0.33 ± 0.03	0.72 ± 0.11	1.15 ± 0.17	1.32 ± 0.12	1.46 ± 0.07	1.48 ± 0.07	1.51 ± 0.08
PRO [mM]	0.30 ± 0.01	0.27 ± 0.02	0.42 ± 0.04	0.53 ± 0.02	0.56 ± 0.02	0.60 ± 0.01	0.60 ± 0.01
MAB [10^−4^ mM]	0.34 ± 0.12	1.02 ± 0.06	1.58 ± 0.16	2.31 ± 0.24	2.66 ± 0.41	3.09 ± 0.60	3.41 ± 0.75
BM [mM]	2.01 ± 0.20	11.61 ± 0.46	16.51 ± 0.85	17.98 ± 0.84	19.41 ± 2.21	18.67 ± 2.49	17.97 ± 1.33
*X* [10^6^ cells/mL]	0.09 ± 0.01	0.58 ± 0.02	0.79 ± 0.05	0.72 ± 0.01	0.47 ± 0.06	0.17 ± 0.03	0.06 ± 0.02
*X*_*d*_ [10^6^ cells/mL]	0.02 ± 0.01	0.05 ± 0.01	0.11 ± 0.01	0.25 ± 0.05	0.58 ± 0.05	0.85 ± 0.12	0.91 ± 0.07

**Table 4:** The subproblems solved by using the multistep PSO-based approach.

Subproblem	Parameters
P1	φ_7_^*^, *K*_*S*_2,7__
P2	φ_8_^*^, *K*_*S*_2,8__
P3	φ_4_^*^, *K*_*S*_3,4__
P4	φ_2_^*^, *K*_*S*_1,2__, *K*_*S*_3,2__
P5	φ_6_^*^, *K*_*S*_2,6__, *K*_*S*_2,9__, φ_9_^*^, *K*_*S*_5,6__
P6	φ_5_^*^, *K*_*S*_4,5__
P7	φ_3_^*^, *K*_*S*_1,3__, *K*_*S*_3,3__
P8	φ_1_^*^, *K*_*S*_1,1__
P9	μ, *k*_*d*_

**Table 5:** Kinetic parameter estimates obtained via the multistep PSO approach.

Kinetic parameters	Estimated values
φ_1_^*^ [pmol/(cell h)]	8.443 × 10^−4^
φ_2_^*^ [pmol/(cell h)]	2.481 × 10^6^
φ_3_^*^ [pmol/(cell h)]	3.968 × 10^5^
φ_4_^*^ [pmol/(cell h)]	1.090 × 10^2^
φ_5_^*^ [pmol/(cell h)]	7.283
φ_6_^*^ [pmol/(cell h)]	3.337 × 10^5^
φ_7_^*^ [pmol/(cell h)]	3.977 × 10^3^
φ_8_^*^ [pmol/(cell h)]	6.697 × 10^−6^
φ_9_^*^ [pmol/(cell h)]	3.261 × 10^4^
*K*_*S*_1,1__ [mM]	8.989 × 10^5^
*K*_*S*_1,2__ [mM]	6.495 × 10^4^
*K*_*S*_1,3__ [mM]	3.723 × 10^4^
*K*_*S*_3,2__ [mM]	7.076 × 10^2^
*K*_*S*_3,3__ [mM]	2.782 × 10^3^
*K*_*S*_3,4__ [mM]	0.019
*K*_*S*_2,6__ [mM]	2.719 × 10^4^
*K*_*S*_2,7__ [mM]	9.324 × 10^3^
*K*_*S*_2,8__ [mM]	0.537
*K*_*S*_2,9__ [mM]	6.683 × 10^5^
*K*_*S*_4,5__ [mM]	1.920 × 10^3^
*K*_*S*_5,6__ [mM]	4.488 × 10^4^
μ	0.043
*k*_*d*_	0.067

**Table 6:** Kinetic parameter estimates obtained by Gao et al. [27] and by Baughman et al. [4], respectively.

Kinetic parameters	Values (Gao et al. [27]) (exponential phase)	Values (Gao et al. [27]) (decline phase)	Values (Baughman et al. [4])
φ_1_^*^ [pmol/(cell h)]	0.008	−0.0033	8.85 × 10^−4^
φ_2_^*^ [pmol/(cell h)]	0.0191	0.0058	1.12 × 10^6^
φ_3_^*^ [pmol/(cell h)]	0.0023	−0.0014	1.1 × 10^5^
φ_4_^*^ [pmol/(cell h)]	0.0081	0.0057	1.97 × 10^−2^
φ_5_^*^ [pmol/(cell h)]	−0.01	0.0056	4.95
φ_6_^*^ [pmol/(cell h)]	−0.011	0.0029	1.34 × 10^5^
φ_7_^*^ [pmol/(cell h)]	0.6429	0.0573	1.36 × 10^3^
φ_8_^*^ [pmol/(cell h)]	0.0046	0.0077	1 × 10^−5^
φ_9_^*^ [pmol/(cell h)]	0.0731	0.0113	1.83 × 10^4^
*K*_*S*_1,1__ [mM]	0.01	0.01	1.63 × 10^5^
*K*_*S*_1,2__ [mM]	0.01	0.01	1.08 × 10^4^
*K*_*S*_1,3__ [mM]	0.01	0.01	1.44 × 10^4^
*K*_*S*_3,2__ [mM]	0.001	0.001	9.64 × 10^2^
*K*_*S*_3,3__ [mM]	0.001	0.001	1.04 × 10^3^
*K*_*S*_3,4__ [mM]	0.001	0.001	5.42 × 10^−2^
*K*_*S*_2,6__ [mM]	0.01	0.01	3.03 × 10^3^
*K*_*S*_2,7__ [mM]	0.01	0.01	6.39 × 10^3^
*K*_*S*_2,8__ [mM]	0.01	0.01	3.73 × 10^−1^
*K*_*S*_2,9__ [mM]	0.01	0.01	3.23 × 10^5^
*K*_*S*_4,5__ [mM]	0.001	0.001	7.42 × 10^3^
*K*_*S*_5,6__ [mM]	0.001	0.001	4.45 × 10^3^
μ	0.0399	0.0399	3.22 × 10^−2^
*k*_*d*_	0.06	0.06	4.99 × 10^−2^

**Table 7:** Monte Carlo parameter means and standard deviation over complete measurement set.

Kinetic parameters	Mean value	Standard deviation
φ_1_^*^ [pmol/(cell h)]	8.127 × 10^−4^	44
φ_2_^*^ [pmol/(cell h)]	2.868 × 10^6^	51
φ_3_^*^ [pmol/(cell h)]	2.441 × 10^5^	63
φ_4_^*^ [pmol/(cell h)]	1.330 × 10^2^	71
φ_5_^*^ [pmol/(cell h)]	7.873	23
φ_6_^*^ [pmol/(cell h)]	3.923 × 10^5^	35
φ_7_^*^ [pmol/(cell h)]	3.157 × 10^3^	93
φ_8_^*^ [pmol/(cell h)]	4.067 × 10^−6^	24
φ_9_^*^ [pmol/(cell h)]	2.991 × 10^4^	36
*K*_*S*_1,1__ [mM]	8.936 × 10^5^	17
*K*_*S*_1,2__ [mM]	7.030 × 10^4^	29
*K*_*S*_1,3__ [mM]	3.684 × 10^4^	5
*K*_*S*_3,2__ [mM]	7.31 × 10^2^	29
*K*_*S*_3,3__ [mM]	2.565 × 10^3^	14
*K*_*S*_3,4__ [mM]	0.026	13
*K*_*S*_2,6__ [mM]	2.295 × 10^4^	87
*K*_*S*_2,7__ [mM]	9.273 × 10^3^	51
*K*_*S*_2,8__ [mM]	0.649	116
*K*_*S*_2,9__ [mM]	6.224 × 10^5^	39
*K*_*S*_4,5__ [mM]	1.814 × 10^3^	44
*K*_*S*_5,6__ [mM]	4.657 × 10^4^	75
μ	0.048	2
*k*_*d*_	0.070	0.73

## References

[1] Slezak D. F., Suárez C., Cecchi G. A., Marshall G., Stolovitzky G. (2010). When the optimal is not the best: parameter estimation in complex biological models. *PLoS ONE*.

[2] Kontoravdi C., Asprey S. P., Pistikopoulos E. N., Mantalaris A. (2007). Development of a dynamic model of monoclonal antibody production and glycosylation for product quality monitoring. *Computers & Chemical Engineering*.

[3] Kontoravdi C., Pistikopoulos E. N., Mantalaris A. (2010). Systematic development of predictive mathematical models for animal cell cultures. *Computers & Chemical Engineering*.

[4] Baughman A. C., Huang X., Sharfstein S. T., Martin L. L. (2010). On the dynamic modeling of mammalian cell metabolism and mAb production. *Computers & Chemical Engineering*.

[5] Batista C. M., Medeiros L. C. S., Eger I., Soares M. J. (2014). MAb CZP-315.D9: an antirecombinant cruzipain monoclonal antibody that specifically labels the reservosomes of *Trypanosoma cruzi* epimastigotes. *BioMed Research International*.

[6] Sautto G., Mancini N., Gorini G., Clementi M., Burioni R. (2013). Possible future monoclonal antibody (mAb)-Based Therapy against arbovirus infections. *BioMed Research International*.

[7] Li F., Vijayasankaran N., Shen A. Y., Kiss R., Amanullah A. (2010). Cell culture processes for monoclonal antibody production. *mAbs*.

[8] Pantazes R. J., Maranas C. D. (2013). MAPs: a database of modular antibody parts for predicting tertiary structures and designing affinity matured antibodies. *BMC Bioinformatics*.

[9] Pavlou A. K., Belsey M. J. (2005). The therapeutic antibodies market to 2008. *European Journal of Pharmaceutics and Biopharmaceutics*.

[10] Dhir S., Morrow K. J., Rhinehart R. R., Wiesner T. (2000). Dynamic optimization of hybridoma growth in a fed-batch bioreactor. *Biotechnology and Bioengineering*.

[11] Giersch C. (2000). Mathematical modelling of metabolism. *Current Opinion in Plant Biology*.

[12] Sidoli F. R., Mantalaris A., Asprey S. P. (2004). Modelling of mammalian cells and cell culture processes. *Cytotechnology*.

[13] Roman M., Selisteanu D., Bobasu E., Sendrescu D. Modeling of culture cells for pharmaceutical industry applications.

[14] Sendrescu D., Bobasu E. Parameter identification of bacterial growth bioprocesses using heuristics for global optimization.

[15] Karnopp D., Rosenberg R. (1974). *System Dynamics: A Unified Approach*.

[16] Thoma J. (1975). *Introduction to Bond Graphs and Their Applications*.

[17] Gawthrop P., Smith L. (1996). *Metamodelling: Bond Graphs and Dynamic Systems*.

[18] Borutzky W. (2009). *Bond Graph Methodology. Development and Analysis of Multidisciplinary Dynamic System Models*.

[19] Dauphin-Tanguy G. (2000). *Les bond graphs*.

[20] Couenne F., Jallut C., Maschke B., Breedveld P. C., Tayakout M. (2006). Bond graph modelling for chemical reactors. *Mathematical and Computer Modelling of Dynamical Systems*.

[21] Heny C., Simanca D., Delgado M. (2000). Pseudo-bond graph model and simulation of a continuous stirred tank reactor. *The Journal of the Franklin Institute*.

[22] Thoma J., Ould Bouamama B. (2000). *Modelling and Simulation in Thermal and Chemical Engineering. A Bond Graph Approach*.

[23] Díaz-Zuccarini V., Rafirou D., LeFevre J., Hose D. R., Lawford P. V. (2009). Systemic modelling and computational physiology: the application of Bond Graph boundary conditions for 3D cardiovascular models. *Simulation Modelling Practice and Theory*.

[24] Selişteanu D., Roman M., Şendrescu D. (2010). Pseudo bond graph modelling and on-line estimation of unknown kinetics for a wastewater biodegradation process. *Simulation Modelling Practice and Theory*.

[25] Roman M., Selisteanu D. (2012). Pseudo bond graph modeling of wastewater treatment bioprocesses. *Simulation*.

[26] Roman M., Selişteanu D. (2012). Enzymatic synthesis of ampicillin: nonlinear modeling, kinetics estimation, and adaptive control. *Journal of Biomedicine and Biotechnology*.

[27] Gao J., Gorenflo V. M., Scharer J. M., Budman H. M. (2007). Dynamic metabolic modeling for a MAb bioprocess. *Biotechnology Progress*.

[28] Petric I., Selimbašić V. (2008). Development and validation of mathematical model for aerobic composting process. *Chemical Engineering Journal*.

[29] Wächter A., Biegler L. T. (2006). On the implementation of an interior-point filter line-search algorithm for large-scale nonlinear programming. *Mathematical Programming: A Publication of the Mathematical Programming Society*.

[30] Schwaab M., Biscaia, E. C., Monteiro J. L., Pinto J. C. (2008). Nonlinear parameter estimation through particle swarm optimization. *Chemical Engineering Science*.

[31] Rezende M. C. A. F., Costa C. B. B., Costa A. C., Maciel M. R. W., Filho R. M. (2008). Optimization of a large scale industrial reactor by genetic algorithms. *Chemical Engineering Science*.

[32] Maraziotis I. A., Dragomir A., Thanos D. (2010). Gene regulatory networks modelling using a dynamic evolutionary hybrid. *BMC Bioinformatics*.

[33] Sîrbu A., Ruskin H. J., Crane M. (2010). Comparison of evolutionary algorithms in gene regulatory network model inference. *BMC Bioinformatics*.

[34] Clerc M., Kennedy J. (2002). The particle swarm-explosion, stability, and convergence in a multidimensional complex space. *IEEE Transactions on Evolutionary Computation*.

[35] Ratnaweera A., Halgamuge S. K., Watson H. C. (2004). Self-organizing hierarchical particle swarm optimizer with time-varying acceleration coefficients. *IEEE Transactions on Evolutionary Computation*.

[36] Sendrescu D. (2013). Parameter identification of anaerobic wastewater treatment bioprocesses using particle swarm optimization. *Mathematical Problems in Engineering*.

[37] Shen B., Liu C., Ye J., Feng E., Xiu Z. (2012). Parameter identification and optimization algorithm in microbial continuous culture. *Applied Mathematical Modelling*.

[38] Zhai J., Ye J., Wang L., Feng E., Yin H., Xiu Z. (2011). Pathway identification using parallel optimization for a complex metabolic system in microbial continuous culture. *Nonlinear Analysis. Real World Applications*.

[39] Yuan J., Zhang X., Zhu X., Feng E., Yin H., Xiu Z. (2014). Modelling and pathway identification involving the transport mechanism of a complex metabolic system in batch culture. *Communications in Nonlinear Science and Numerical Simulation*.

[40] Ye J., Zhang Y., Feng E., Xiu Z., Yin H. (2012). Nonlinear hybrid system and parameter identification of microbial fed-batch culture with open loop glycerol input and pH logic control. *Applied Mathematical Modelling*.

[41] Li X., Zhang S., Xiu Z., Feng E. (2012). Parameter identification model with the control term in batch anaerobic fermentation. *Applied Mechanics and Materials*.

[42] Liao Z., Mei C. (2011). Estimation of biochemical variables using quantumbehaved particle swarm optimization (QPSO)-trained radius basis function neural network: a case study of fermentation process of L-glutamic acid. *African Journal of Biotechnology*.

[43] Kennedy J., Eberhart R. C. Particle swarm optimization.

[44] Gambhir A., Korke R., Lee J., Fu P. C., Europa A., Hu W. S. (2003). Analysis of cellular metabolism of hybridoma cells at distinct physiological states. *Journal of Bioscience and Bioengineering*.

[45] Bonarius H. P. J., Hatzimanikatis V., Meesters K. P. H., de Gooijer C. D., Schmid G., Tramper J. (1996). Metabolic flux analysis of hybridoma cells in different culture media using mass balances. *Biotechnology and Bioengineering*.

[46] Follstad F. D., Balcarcel R. R., Stephanopoulos G., Wang D. I. C. (1999). Metabolic flux analysis of hybridoma continuous culture steady state multiplicity. *Biotechnology and Bioengineering*.

[47] Frame K. K., Hu W.-S. (1991). Kinetic study of hybridoma cell growth in continuous culture. I. A model for non-producing cells. *Biotechnology and Bioengineering*.

[48] Provost A., Bastin G., Agathos S. N., Schneider Y. J. (2006). Metabolic design of macroscopic bioreaction models: application to Chinese hamster ovary cells. *Bioprocess and Biosystems Engineering*.

[49] Bastin G., Dochain D. (1990). *On-Line Estimation and Adaptive Control of Bioreactors*.

[50] Dochain D. (2008). *Automatic Control of Bioprocesses*.

[51] Hasanvand H., Zad B. B., Mozafari B., Maskani H. (2011). Fuzzy logic controller design based SVC for improving power system damping. *International Review of Automatic Control*.

[52] Amlashi Y. B., Afrakhte H. (2011). Determination of wind plant output capacity using discrete Markov chains and PSO methods in comparison with FCM. *International Review on Modelling & Simulations*.

[53] Eberhart R. C., Shi Y. Comparing inertia weights and constriction factors in particle swarm optimization.

[54] Shi Y., Eberhart R. C. Empirical study of particle swarm optimization.

[55] Tetko I. V., Livingstone D. J., Luik A. I. (1995). Neural network Studies. 1. Comparison of overfitting and overtraining. *Journal of Chemical Information and Computer Sciences*.

[56] Culshaw R. V., Ruan S., Webb G. (2003). A mathematical model of cell-to-cell spread of HIV-1 that includes a time delay. *Journal of Mathematical Biology*.

[57] Niculescu S.-I., Morărescu C.-I., Michiels W., Gu K., I. Queinnec, Tarbouriech S., Garcia G. (2007). Geometric ideas in the stability analysis of delay models in biosciences. *Biology and Control Theory: Current Challenges*.

